# Supplementary material for: Hybrid Models and Biological Model Reduction with PyDSTool
Source: PLoS Comput Biol. 2012 Aug 9;8(8):e1002628. doi: 10.1371/journal.pcbi.1002628 (PMC3415397; doi:10.1371/journal.pcbi.1002628)
Supplement: Text S4 — Complete source code for the PyDSTool package (version 0.88.120504). Includes API documentation and help files linking to web pages. This file is identical to the current public release on Sourceforge.net. (ZIP) [file pcbi.1002628.s004.zip › PyDSTool/html/PyDSTool.PyCont.Continuation.Continuation-class.html]

xml version="1.0" encoding="ascii"?


PyDSTool.PyCont.Continuation.Continuation


| Home | Trees | Indices | Help | PyDSTool |
| --- | --- | --- | --- | --- |

Package PyDSTool ::
Package PyCont ::
Module Continuation ::
Class Continuation

[hide private]

[frames] | no frames]

# Class Continuation

source code

```
object --+
         |
        Continuation
```

Known Subclasses:
:   - EquilibriumCurve
    - , FixedPointCurve
    - , FoldCurve
    - , HopfCurveOne
    - , HopfCurveTwo
    - , LimitCycleCurve
    - , UserDefinedCurve

---

Abstract continuation class

Children: EquilibriumCurve, FoldCurve, HopfCurveOne, HopfCurveTwo,
LimitCycleCurve


|  |  |
| --- | --- |
| Instance Methods | [hide private] |

|  |  |
| --- | --- |
| \_\_init\_\_(self, model, gen, automod, plot, args=None)  x.\_\_init\_\_(...) initializes x; see x.\_\_class\_\_.\_\_doc\_\_ for signature | source code |

|  |  |
| --- | --- |
| \_\_copy\_\_(self) | source code |

|  |  |
| --- | --- |
| \_\_deepcopy\_\_(self, memo=None, \_nil=`[``]`) | source code |

|  |  |
| --- | --- |
| reset(self, args=None)  Resets curve by setting default parameters and deleting solution curve. | source code |

|  |  |
| --- | --- |
| update(self, args)  Update parameters for Continuation. | source code |

|  |  |
| --- | --- |
| \_preTestFunc(self, X, V) | source code |

|  |  |
| --- | --- |
| \_createTestFuncs(self)  Creates processors and test functions for Continuation class. | source code |

|  |  |
| --- | --- |
| \_system(self, X) | source code |

|  |  |
| --- | --- |
| \_systemjac(self, x0, ind=None) | source code |

|  |  |
| --- | --- |
| \_systemjac\_withpars(self, x0, ind=None) | source code |

|  |  |
| --- | --- |
| \_systemuser(self, X)  Calls self.\_userfunc, which is assumed to return an array of RHS values for the relevant (possibly subset of) variables. | source code |

|  |  |
| --- | --- |
| \_systemjacuser(self, x0, ind=None)  Calls self.\_userjac, which is assumed to return an array of [Jac\_x, Jac\_p]. | source code |

|  |  |
| --- | --- |
| \_checkForBifPoints(self) | source code |

|  |  |
| --- | --- |
| exportGeomview(self, coords=None, filename=`'``geom.dat``'`) | source code |

|  |  |
| --- | --- |
| display(self, coords=None, dirs=None, origin=None, figure=None, axes=None, stability=False, domain=False, init\_display=True, points=True, \*\*plot\_args)  Plot curve in coordinates specified by coords. | source code |

|  |  |
| --- | --- |
| \_savePointInfo(self, loc)  Created a function for this since it needs to be called both in \_compute and when a bifurcation point is found. | source code |

|  |  |
| --- | --- |
| \_MoorePenrose(self, X, V) | source code |

|  |  |
| --- | --- |
| \_Natural(self, X, V) | source code |

|  |  |
| --- | --- |
| \_Keller(self, X, V) | source code |

|  |  |
| --- | --- |
| \_compute(self, x0=None, v0=None, direc=1)  Continuation using Moore-Penrose method (called by forward and backward methods) | source code |

|  |  |
| --- | --- |
| forward(self)  Computes forward along curve from initpoint if this is the first run. | source code |

|  |  |
| --- | --- |
| backward(self)  Computes backward along curve from initpoint if this is the first run. | source code |

|  |  |
| --- | --- |
| testdomain(self, ic=None, ind=0, direc=1) | source code |

|  |  |
| --- | --- |
| testdomaingrid(self, ic=None, coords=`(``'``y``'``,` `'``theta``'``)`, Dx=None, Dy=None, step=2) | source code |

|  |  |
| --- | --- |
| testdomaintangrid(self, Dx, Dy, ic=None, step=2) | source code |

|  |  |
| --- | --- |
| \_curveToPointset(self) | source code |

|  |  |
| --- | --- |
| getSpecialPoint(self, label1, label2=None)  Gets a point on the curve with name specified by label1 and label2. | source code |

|  |  |
| --- | --- |
| computeEigen(self) | source code |

|  |  |
| --- | --- |
| cleanLabels(self) | source code |

|  |  |
| --- | --- |
| info(self) | source code |

|  |  |
| --- | --- |
| \_\_repr\_\_(self)  str(x) | source code |

|  |  |
| --- | --- |
| \_\_str\_\_(self)  str(x) | source code |

**Inherited from `object`**:
`__delattr__`,
`__getattribute__`,
`__hash__`,
`__new__`,
`__reduce__`,
`__reduce_ex__`,
`__setattr__`


|  |  |
| --- | --- |
| Properties | [hide private] |

**Inherited from `object`**:
`__class__`


|  |  |
| --- | --- |
| Method Details | [hide private] |

|  |  |
| --- | --- |
| \_\_init\_\_(self, model, gen, automod, plot, args=None)  *(Constructor)* | source code |

x.\_\_init\_\_(...) initializes x; see x.\_\_class\_\_.\_\_doc\_\_ for
signature

Overrides: object.\_\_init\_\_: *(inherited documentation)*

|  |  |
| --- | --- |
| \_createTestFuncs(self) | source code |

```
Creates processors and test functions for Continuation class.

Note:  In the following list, processors are in PyCont.Bifpoint
and test functions are in PyCont.TestFunc.

Point type (Processor): Test Function(s)
----------------------------------------

BP (BranchPoint): Branch_Det
```

|  |  |
| --- | --- |
| display(self, coords=None, dirs=None, origin=None, figure=None, axes=None, stability=False, domain=False, init\_display=True, points=True, \*\*plot\_args) | source code |

```
Plot curve in coordinates specified by coords.

Inputs:

    coords -- tuple of coordinates (None defaults to the first free
        parameter and the first state variable)
    dirs -- tuple of coordinate directions IF coord is not in regular coords
    origin -- Useful if want affine coordinates
```

|  |  |
| --- | --- |
| \_savePointInfo(self, loc) | source code |

Created a function for this since it needs to be called both in
\_compute and when a bifurcation point is found. It will have conditional
statements for saving of Jacobian and eigenvalues, as well as other
possible tidbits of information.

|  |  |
| --- | --- |
| \_compute(self, x0=None, v0=None, direc=1) | source code |

Continuation using Moore-Penrose method (called by forward and
backward methods)

NOTE: For codimension 2 curves, CorrFunc is the augmented system
consisting of sysfunc with testfunc associated with the curve given by
sysfunc. When you call CorrFunc, it calls sysfunc.PreTestFunc to
calculate the jacobian that testfunc needs. THUS, sysfunc jacobians are
computed and stored in sysfunc. Now, if you have test functions that
require jacobian information from sysfunc and CorrFunc, I only call
CorrFunc.PreTestFunc. BUT, it still works because CorrFunc was called
just previous, thereby saving sysfunc jacobians that are needed by the
test functions that rely on sysfunc jacobians. Understand? Good, cuz I
barely do. I'm going to try and alleviate this confusion. Wish me
luck... By the way, the example of this is in computing ZH points. They
require test functions for a codimension 1 curve while continuing a
codimension 2 curve.

|  |  |
| --- | --- |
| forward(self) | source code |

Computes forward along curve from initpoint if this is the first run.
Otherwise, it computes forward along curve from the last point on the
saved solution sol. The new curve is appended to the end of sol.

|  |  |
| --- | --- |
| backward(self) | source code |

Computes backward along curve from initpoint if this is the first run.
Otherwise, it computes backward along curve from the first point on the
saved solution sol. The new curve is appended to the front of sol.

|  |  |
| --- | --- |
| getSpecialPoint(self, label1, label2=None) | source code |

```
Gets a point on the curve with name specified by label1 and
label2.

   Inputs:

       label1 -- string
       label2 -- string

   Output:

       x -- Point with specified name (type Point)

   If label2 is None, then label1 needs to be the name of the
   point.  In this case, the point type should be apparent
   from the name (i.e. by stripping off digits from the
   right).

   If label2 is not None, then label1 should be the point type
   and label2 the point name.

   For example, the following two function calls are
   equivalent:

       getSpecialPoint('LP3')
       getSpecialPoint('LP','LP3')
```

|  |  |
| --- | --- |
| \_\_repr\_\_(self)  *(Representation operator)* | source code |

str(x)

Overrides: object.\_\_repr\_\_: *(inherited documentation)*

|  |  |
| --- | --- |
| \_\_str\_\_(self)  *(Informal representation operator)* | source code |

str(x)

Overrides: object.\_\_str\_\_: *(inherited documentation)*

  


| Home | Trees | Indices | Help | PyDSTool |
| --- | --- | --- | --- | --- |

|  |  |
| --- | --- |
| Generated by Epydoc 3.0.1 on Fri May 4 15:24:07 2012 | http://epydoc.sourceforge.net |
